# Modifying feeding protocols in critically ill patients based on a predictive model of feeding intolerance: protocol for a multicenter cluster randomized controlled trial (the mNEED study)

**DOI:** 10.3389/fmed.2025.1649983

**Published:** 2025-11-06

**Authors:** Youquan Wang, Yanhua Li, Ying Chen, Yuhan Zhang, Xuewen Feng, Dongfang Lv, Yumeng Chen, Xu Bi, Yongfu Yu, Yuwei Peng, Hongxiang Li, Dong Zhang

**Affiliations:** 1 Department of Critical Care Medicine, The First Hospital of Jilin University, Changchun, China; 2 Key Laboratory of Public Health Safety of Ministry of Education, Department of Biostatistics, Fudan University, Shanghai, China; 3 Key Laboratory for Health Technology Assessment, Shanghai, China; 4 School of Public Health, National Commission of Health, Shanghai, China

**Keywords:** critical care, feeding intolerance, clinical outcome, ultrasound, cluster-randomized trial, nutrition therapy

## Abstract

**Objectives:**

It remains unclear whether the modified nutrition protocol for critically ill patients (mNEED), based on the NOFI predictive model for feeding intolerance (FI), can reduce the incidence of FI and subsequently improve clinical outcomes.

**Design:**

This is a multicenter, cluster-randomized controlled trial.

**Setting:**

Ninety intensive care units (ICUs) across China.

**Participants:**

A total of 2,250 critically ill patients initiating early enteral nutrition who meet the inclusion criteria.

**Intervention:**

In addition to standard care, centers randomized to the intervention arm will implement nutritional therapy strictly according to the mNEED protocol.

**Primary outcome measure:**

The primary outcome is the incidence of FI within the first 7 days of ICU admission. Secondary outcomes and process measures will also be reported. An intention-to-treat analysis will be conducted for the primary outcome to ensure the robustness of the findings.

**Ethics and dissemination:**

This study has been approved by the Ethics Committee of the First Hospital of Jilin University (original version: 24k004-001; revised version: 24k004-002) and by the ethics committees of all participating sites. Results will be disseminated through peer-reviewed publications and conference presentations.

**Trial registration:**

This trial has been registered at the Chinese Clinical Trial Registry (ChiCTR2400081581): https://www.chictr.org.cn/showproj.html?proj=219477 and at ClinicalTrials.gov (NCT06827275): https://clinicaltrials.gov/study/NCT06827275.

## 1 Introduction

Nutritional support is critically important for patients in the intensive care unit (ICU). Major international evidence-based guidelines consistently recommend the provision of early, targeted nutritional therapy for critically ill patients (1, 2). However, cluster randomized controlled trials (cRCTs) evaluating the impact of guideline implementation on early targeted nutrition have not demonstrated patient benefit (3, 4). In 2022, Ke et al. proposed the Nutrition Evidence-based feeding guidelines for critically ill patients (NEED), which offer a specific feeding protocol for nutritional therapy in critical illness (4). The results suggested that the NEED group initiated enteral nutrition (EN) earlier and had reduced overall parenteral nutrition (PN) use.

Nevertheless, analysis of the study data revealed that the mean time to EN initiation in the NEED group was 1.2 days, and only about 60% of patients achieved 70% of target energy via EN within 72 h. The remaining 40% of patients met the definition of feeding intolerance (FI) (5). Notably, this study did not report the incidence of gastrointestinal (GI) complications such as diarrhea or gastric retention. Given the substantial number of patients with suboptimal EN delivery, it can be inferred that nearly half of the patients in the NEED group may have met the diagnostic criteria for FI, with no significant difference compared to the control group. This may indicate that the NEED protocol did not reduce—and may have even increased—the incidence of FI. In the acute phase of critical illness, the occurrence of FI is often one of the most clinically concerning events for ICU physicians.

Feeding intolerance may result in feeding interruption or delay, thereby hindering the successful implementation of EN and its associated benefits. This can lead to delayed energy target achievement, development of malnutrition (6), prolonged hospital stays (7), and even increased mortality (8, 9). Studies have shown that persistent FI during the first week of ICU admission is an independent risk factor for 28-day and 90-day mortality (9). Therefore, optimizing feeding strategies to reduce the occurrence of FI holds significant clinical importance.

In our previous study, we developed a predictive model for FI in critically ill patients—NOFI—which incorporates primary diagnosis, the Acute Physiology and Chronic Health Evaluation II (APACHE II) score, and Acute Gastrointestinal Injury (AGI) grade to predict the individual risk of FI (10). This model demonstrated good discrimination, calibration, and clinical utility. Based on this model, we aim to modify the NEED protocol (modified NEED, or mNEED) to reduce the occurrence of FI while still meeting the nutritional needs of critically ill patients.

The objective of this study is to evaluate the impact of the NOFI-based modified NEED protocol on patient outcomes. A cluster-randomized design will be adopted, in which participating ICUs will be randomly assigned to either the mNEED group or the standard guideline-based group. We hypothesize that the mNEED protocol may reduce the incidence of FI and potentially improve the prognosis of critically ill patients compared to standard guideline-based care.

## 2 Methods and analysis

### 2.1 Study design

This is a multicenter cluster randomized controlled trial conducted by the Chinese Critical Care Nutrition Trials Group (ChiCTR2400081581^1^; NCT06827275^2^). The trial protocol was developed in accordance with the Standard Protocol Items: Recommendations for Interventional Trials (SPIRIT) statement (11). The final version of the plan is 5.0, with the date being 16 December 2024.

### 2.2 Study population

All patients will be screened consecutively during routine clinical care: every individual admitted to the participating hospitals’ ICUs will be evaluated for eligibility. Enrollment in the trial will proceed only after written informed consent has been obtained from the patient or a legally authorized representative.

#### 2.2.1 Inclusion criteria (for patients)

1. Aged ≥ 18 years;
2. Presence of one or more organ system failures within 24 h of ICU admission (SOFA score ≥ 2);
3. Expected ICU stay > 48 h;
4. Inability to take oral nutrition;
5. No contraindications to enteral nutrition (EN).

#### 2.2.2 Exclusion criteria (for patients)

1. Received EN therapy within the past 3 days;
2. Grade IV acute gastrointestinal injury (AGI IV);
3. Receiving palliative care with expected death within 48 hours;
4. Pregnancy;
5. Long-term use of corticosteroids or other immunosuppressants;
6. Undergoing radiotherapy or chemotherapy for malignant diseases;
7. Participation in another clinical trial.

#### 2.2.3 ICU inclusion and exclusion criteria

To minimize the impact of inter-institutional differences in medical practice on clinical outcomes, the following criteria were established for participating ICUs.

##### 2.2.3.1 ICU inclusion criteria

1. ICUs within secondary or tertiary hospitals;
2. ICUs capable of delivering intensive care and monitoring FI;
3. ICU types including emergency, medical, surgical, neurosurgical, or general ICUs.

##### 2.2.3.2 ICU exclusion criteria

1. ICUs that decline participation;
2. ICUs that fail ethical review approval;
3. Pediatric ICUs.

### 2.3 Sample size calculation

Sample size calculation was performed using PASS software. The primary clinical outcome was the difference in the incidence of FI between the two groups. Based on previous studies, the incidence of FI among critically ill patients in the control group was assumed to be 0.5. According to the clinical decision curve analysis from the predictive model for FI (NOFI) (10), the net benefit at a threshold probability of approximately 50% was 0.257, indicating that around 25.7 patients out of every 100 would benefit from the intervention. Therefore, it was assumed that the mNEED protocol (intervention group) would reduce the incidence of FI by 25%, resulting in an expected FI rate of 0.375 in the intervention group [0.5 × (1–0.25) = 0.375]. We set the intraclass correlation coefficient (ICC) to 0.10 by analogy to the NEED trial (4), a total of 45 clusters per group were planned, with an ICC of 0.1. Using a two-sided alpha of 0.05 and a statistical power of 90% (1–β = 0.9), the required number of participants per cluster was calculated to be 25. Thus, the total sample size was 45 clusters × 25 participants × 2 groups = 2,250 participants. We pre-specified ICC sensitivity analyses, which indicated that the planned configuration is robust to plausible ICC and cluster-size variability; detailed results are provided in Supplementary Tables 3, 4.

### 2.4 Randomization and study intervention

#### 2.4.1 Randomization

This study employed a multicenter, stratified block-randomized controlled trial with cluster allocation at the ICUs level. A total of 108 ICUs were enrolled, including 102 tertiary general hospital ICUs and 6 other-level ICUs, as of 15 June 2025. Stratified randomization was performed by first stratifying ICUs according to their level (tertiary general hospital/others), followed by generating block randomization sequences within each stratum using R software (version 4.4.1). Clusters were allocated in a 1:1 ratio to either the intervention group or control group. Finally, 54 ICUs were assigned to each group. Due to the nature of the intervention, a partial blinding approach was adopted: participating patients, center implementers, and outcome assessors were aware of group allocation, while members of the endpoint adjudication committee remained blinded to allocation status.

#### 2.4.2 Blinding

Participants or their legal representatives will be completely blinded to group allocation. Healthcare providers will only be aware of the feeding protocol used in their own center and will not be informed of group assignments in other centers. Outcome assessments will be conducted by a dedicated research team that is not involved in the implementation of the feeding protocols and is unaware of the grouping of these patients. For the primary outcome, after the study is completed, GI symptoms and daily feeding data recorded in the database will be reviewed and consolidated by the research team at the coordinating center to perform a unified evaluation. Statistical analyses will also be conducted by researchers at the coordinating center. Emergency unblinding will be permitted in cases of safety concerns or when significant differences in outcomes between groups are observed.

#### 2.4.3 Interventional arms

According to the randomization scheme, all centers will be assigned to either the intervention group or the control group in a 1:1 ratio before patient recruitment begins. Table 1 shows the schedule of enrollment, interventions, and assessments based on the SPIRIT 2013 statement (11). Detailed reporting checklists are provided in the Supplementary Table 1 (SPIRIT 2013 checklist) and Supplementary Table 2 (CONSORT–Cluster checklist) (12).

**TABLE 1:** Schedule of enrollment, interventions, and assessments.

	Study period						
	Enrollment	Allocation	Post-allocation				Close-out
Timepoint	0–48 h	0–48 h	Day 1	Day 7	Day 28	Day 90	T_*E*_
Enrollment:							
Eligibility screen	X	–	–	–	–	–	–
Informed consent	X	–	–	–	–	–	–
Allocation	–	X	–	–	–	–	–
Interventions:	–	–	↔			–	–
(mNEED group)	–	–	↔			–	–
(Control group)	–	–	–	–	–	–	–
Assessments:	–	–	–	–	–	–	–
(Baseline characteristics)	X	X	–	–	–	–	–
(Muscle ultrasound assessment)	–	–	X	–	X	–	–
(Laboratory tests)	–	–	↔			–	–
(Therapies and medication use)	–	–	↔			–	–
(Critical illness and nutrition-related scores)	–	–	↔			–	–
(Nutrition delivery)	–	–	↔			–	–
(Gastrointestinal symptoms]	–	–	↔			–	–
(Rehabilitation therapy)	–	–	↔			–	–
(Clinical outcomes)	–	–	↔				–
(SF-36 and EQ-5D-5L)	–	–	–	–	X	X	
(Adverse events)	–	–	–	–	–	–	X

##### 2.4.3.1 Arm#1 mNEED group

If a center is randomized to the intervention group, training on the implementation of the mNEED nutrition protocol and ultrasound assessment will be conducted before enrolling the first patient. The mNEED protocol includes several modifications to the original NEED protocol:

1. For patients with AGI ≤ III, the risk of feeding intolerance (FI) is predicted using the NOFI model. Based on the predicted probabilities (< 30%, 30%–85%, and > 85%), different feeding strategies are adopted according to the threshold values identified from the NOFI decision curve analysis.
2. The initial feeding rates have been reduced overall. Instead of the original rates of 25 mL/h for AGI < I and 15 mL/h for AGI II–III, the revised rates are: 20 mL/h for < 30% predicted FI risk, 15 mL/h for 30%–50%, and 10 mL/h for 50%–85%.
3. For AGI ≤ III patients with a predicted FI probability > 85%, early EN (EEN) is deferred, and re-evaluation is performed after 12 h.
4. The FI assessment interval has been extended from 4 to 6 h to every 12 h. Additionally, when the FIS (Feeding Intolerance Score) is 0, the incremental feeding volume has been reduced from 10 to 5 mL/h. The rationale for both adjustments is consistent with the overall strategy: to avoid overly rapid feeding advancement during the acute phase, which increases the risk of FI and early overfeeding.
5. A new item has been added to limit energy provision to no more than 70% of the target caloric intake during the first 3 days, in accordance with ESPEN guidelines, to minimize the risk of early overfeeding.
6. The decision to initiate supplemental parenteral nutrition (SPN) is based on evaluating the patient’s nutritional needs curve. The objective is to align nutritional delivery as closely as possible with this curve, ensuring adequate energy provision while avoiding overfeeding.

To monitor whether enrolled patients are managed in accordance with the study protocol, research staff at each participating center are required to complete a feeding record form for every patient until ICU discharge. This form includes detailed components of the FIS, with evaluations performed every 12 h. It records the FIS values and corresponding adjustments in the feeding rate. Upon study completion, electronic versions of the FIS record forms for all patients will be collected via email.

**FIGURE 1:**
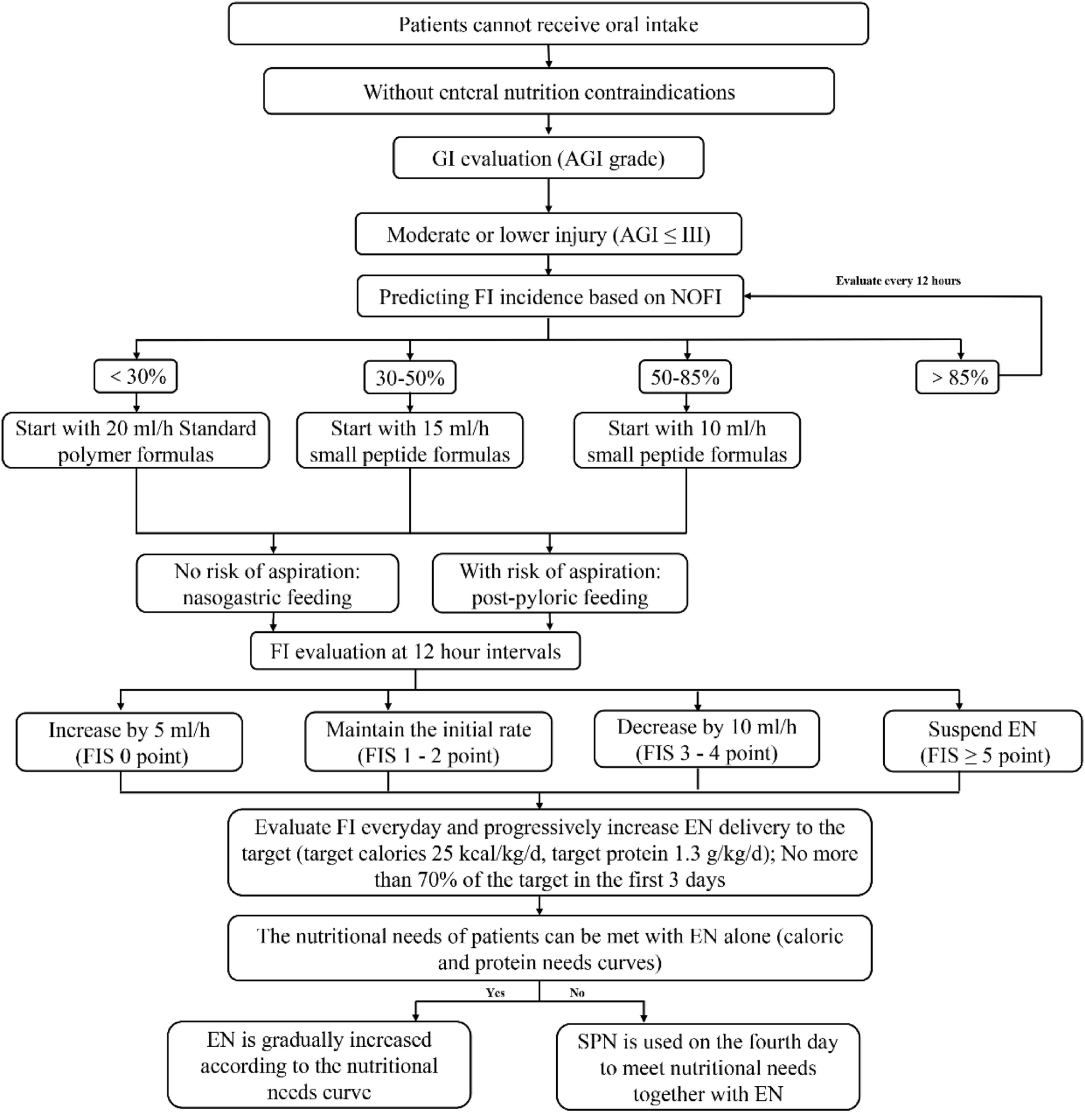
Modified nutrition protocol for critically ill patients (mNEED) nutrition implementation process.

The detailed implementation flow of the mNEED protocol is presented in Figure 1. To facilitate the use of NOFI, we have developed a publicly accessible web tool for quick calculation of the predicted FI risk: https://youquan.shinyapps.io/shiny_app/. In addition, a printable bedside nomogram was provided so clinicians can apply NOFI at the bedside when the web tool is unavailable.

The management of GI adverse events follows the protocol established in the original NEED study (4). Each intervention center will receive a poster displaying the feeding protocol and GI event management flowchart, to be posted in the ICU ward for ease of reference and implementation.

##### 2.4.3.2 Arm#2 Control group

Centers randomized to the control group will follow general nutrition practices as recommended by the ESPEN guidelines. Prior to enrolling the first patient, training on the interpretation of the ESPEN guidelines and ultrasound assessment will also be provided. The control group does not follow a specified feeding protocol, but adheres to guideline-based principles, including: providing no more than 70% of estimated energy expenditure (EE) in the first 3 days; advancing to 80%–100% of EE by days 4–7; and initiating SPN on days 4–7 if EN alone is insufficient to meet nutritional needs.

Management of GI adverse events in the control group is consistent with that of the intervention group. Control group centers will also receive dedicated posters for reference and implementation.

##### 2.4.3.3 Ultrasound training for both groups

Ultrasound training will be provided to both intervention and control groups prior to the enrollment of the first patient at each center. All ultrasound training will be delivered by certified trainers from the Chinese Critical Care Ultrasound Study Group (CCUSG).

All participating centers involved in muscle ultrasound measurements are required to preserve each measurement as an ultrasound image. These images should be retained locally during the study and uploaded collectively after study completion. Standardized measurement protocols and instructional videos are available via the following platforms: Bilibili^3^ or YouTube^4^.

To address potential operator-dependent variability, we will account for it when reporting ultrasound outcomes, including inter-rater reliability metrics on a centrally read subset.

#### 2.4.4 Protocol deviations

##### 2.4.4.1 mNEED group

Major deviations [exclude from per-protocol set (PPS)]: Initial EN not delivered per protocol (speed or formula at start not matching protocol specification), or violation of the EN protocol more than once, as documented in the uploaded electronic feeding record.

Minor deviations (retain in PPS): Violation of the EN protocol no more than once.

##### 2.4.4.2 Control group

Major deviations: The EN speed reached 25 kcal/kg/day within 24 h of starting EN.

Minor deviations: Initial EN amount > 50% of 25 kcal/kg/day at start of EN.

### 2.5 General management

All other aspects of patient management will be conducted according to standard clinical practice at each participating ICU. This includes, but is not limited to, monitoring of vital signs, laboratory testing, fluid resuscitation, mechanical ventilation, and vasopressor support, as deemed necessary by the attending medical team. No interventions will be applied to any aspect of care other than nutritional support. All concomitant treatments will be determined by the responsible clinicians and documented in the patient’s medical records.

### 2.6 Study outcomes and their measures

#### 2.6.1 Primary outcome definition and measures

Occurrence of FI during the acute phase of critical illness (within the first 7 days of ICU admission) (5). FI is defined as either:

- Intolerance to EN due to any clinical reason [e.g., vomiting (any visible regurgitation of gastric content irrespective of the amount), high gastric residual volume (a single volume exceeds 200 ml), diarrhea (having three or more loose or liquid stools per day with a stool weight greater than 200–250 g/day (or greater than 250 ml/day)], GI bleeding [(any bleeding into the GI tract lumen, confirmed by macroscopic presence of blood in vomited fluids, gastric aspirate or stool), etc.,], or
- FI should be considered present if at least 20 kcal/kg BW/day via enteral route cannot be reached within 72 h of feeding attempt or if enteral feeding has to be stopped for whatever clinical reason.

### 2.6.1.1 Sensitivity analysis using an alternative definition (EFI)

To assess the robustness of the primary outcome, a pragmatic alternative definition of enteral feeding intolerance (EFI) will also be applied in sensitivity analyses. EFI is defined as a clinician’s decision to reduce the prescribed amount of EN specifically because features of gastrointestinal dysfunction appeared during feeding (13). Results derived from this EFI definition will be reported as sensitivity analyses and interpreted only as supportive evidence for the primary outcome, not as separate primary or secondary outcomes.

Since the diagnosis of FI is not completely objective (14), FI was independently adjudicated by two blinded outcome evaluators (Youquan Wang and Yanhua Li) using a de-identified dataset containing only the prespecified variables (binary gastrointestinal-symptom indicators and 72-h EN delivery data). The evaluators were blinded to treatment allocation and site identities. Dong Zhang collated the two assessments; in case of disagreement, he served as the third reviewer and made the final determination. In the final outcome report, all FI cases will be categorized and reported per component as: (i) FI due to gastrointestinal symptom(s), (ii) FI due to inadequate EN delivery, or (iii) both criteria met.

#### 2.6.1.2 Handling of incomplete FI assessments

(1). If a patient experiences an ICU disposition (death or discharge/transfer) within 7 days of ICU admission, the FI window spans ICU admission to the time of disposition. (2). If no prespecified gastrointestinal (GI) symptom is documented and the patient has an ICU disposition within 72 h of EN initiation such that FI cannot be fully ascertained, the primary analysis will classify the patient as no FI.

Adjusted FI (aFI) sensitivity definition. For the scenario described in rule (2) above, we prespecify an adjusted FI endpoint (aFI) for sensitivity analyses:

If any EN reduction is recorded, classify as FI

If no EN reduction is recorded, classify as no FI.

#### 2.6.2 Secondary outcome definition and measures

1. Achievement of nutritional targets: Receiving at least 80% of the estimated nutritional goals by day 7 after ICU admission
2. (defined as energy intake ≥ 25 kcal/kg/day and protein intake ≥ 1.3 g/kg/day)
3. 28-day mortality: Assessed on day 28 after ICU admission
4. 90-day mortality: Assessed on day 90 after ICU admission
5. Health-related quality of life (HRQoL) measured by SF-36 (Short Form-36 Health Survey): Assessed on days 28 and 90 after ICU admission
6. Health-related quality of life measured by EQ-5D-5L (EuroQol 5 Dimensions 5 Levels): Assessed on days 28 and 90 after ICU admission
7. Diaphragm thickness: Measured on day 7 after ICU admission
8. Quadriceps muscle thickness: Measured on day 7 after ICU admission
9. Rectus femoris cross-sectional area: Measured on day 7 after ICU admission
10. Upper limb muscle thickness: Measured on day 7 after ICU admission
11. New infections in the ICU: Defined as infections newly acquired ≥ 48 hours after ICU admission
12. ICU length of stay
13. Ventilator-free days: Ventilator-free days within 28 days in the ICU

#### 2.6.3 Process measures

To evaluate the implementation and adherence to nutritional strategies in both groups, the following process measures will be collected:

1. Time from ICU admission to initiation of feeding: to assess the timeliness of nutritional support initiation.
2. Time from study enrollment to initiation of feeding: to evaluate the efficiency of implementing the assigned nutritional protocol after enrollment.
3. Percentage of patients fed within 24 h of ICU admission: to assess compliance with early feeding recommendations.
4. Mean daily energy delivered (kcal/kg/day): to quantify caloric intake and assess nutritional adequacy.
5. Mean daily protein delivered (g/kg/day): to quantify protein intake throughout the ICU stay.
6. Completeness of the feeding record forms in the intervention group: to assess fidelity to the mNEED protocol, reflecting whether nutritional interventions were implemented as per protocol.
7. Daily caloric and protein intake in the control group: to determine whether the feeding pattern followed the progressive strategy recommended by the ESPEN guidelines (e.g., not exceeding 70% of energy expenditure in the first 3 days, and gradual advancement thereafter).
8. Use of supplemental protein on day 4 after ICU admission (e.g., oral protein supplements or intravenous amino acids): to evaluate whether both groups followed guideline-recommended escalation of protein delivery, ensuring protein targets were progressively achieved.

#### 2.6.4 Duration of treatment and follow-up

The study intervention will continue for 28 days following ICU admission, unless the patient is discharged from the ICU, discharged from the hospital, or dies earlier. All randomized patients will be followed for 90 days after ICU admission, unless death occurs first. If a patient remains hospitalized on day 90, follow-up will be conducted and outcomes will be recorded based on the patient’s status on day 90.

#### 2.6.5 Reasons for withdrawal/discontinuation

If any serious adverse events related to the study occur during the trial, early withdrawal from the study will be permitted. In addition, if a patient or their legal representative requests withdrawal from the trial for any reason, early withdrawal will be allowed without affecting the patient’s future medical care from the investigators or study site. Efforts will be made to follow up all randomized patients to the extent permitted by their consent.

#### 2.6.6 Intervention fidelity, contamination, and exposure

Fidelity: Using the electronic feeding logs and CRFs, we will quantify protocol fidelity in the mNEED arm as the proportion of patients who completed every protocol-mandated EN dose adjustment at each scheduled assessment (per protocol rules). This will be summarized at patient and ICU (cluster) levels.

Contamination (control arm): Each control ICU will be contacted to confirm whether NOFI (the FI prediction model) was used to guide EN decisions. Any reported use of NOFI to guide EN in the control arm will be recorded as intervention contamination for that ICU.

Exposure metrics. To contextualize delivery of nutrition support, we will report: (i) time to EN initiation from ICU admission; and (ii) duration (days) under protocol-managed nutrition. These process measures are not clinical endpoints; they will be summarized descriptively and compared between arms.

### 2.7 Data collection

A secure web-based electronic data capture (EDC) system, provided by Unimed Scientific Inc. (Wuxi, China), will be used for data collection and storage. Each participating center will be assigned a single user account, and one designated staff member will be responsible for data entry. The trial management committee will oversee user authorization. Training on the use of the EDC system will be provided by the database vendor in collaboration with the coordinating center of the CCCNTG.

#### 2.7.1 Data analysis

All statistical analyses will be conducted in accordance with a pre-specified statistical analysis plan. The primary analysis will follow the intention-to-treat (ITT) principle, with adjustment for clustering. The primary outcome (FI, binary) will be analyzed using a generalized linear mixed model (GLMM, logit link) with a random intercept for ICU/cluster and fixed effects for treatment and the pre-specified covariates (APACHE II score, AGI grade, primary diagnosis). To guard against model mis-specification and unequal cluster sizes, cluster-robust (sandwich) standard errors will be used for inference with between-cluster degrees of freedom; if convergence or small-sample issues arise, a wild cluster bootstrap-t procedure will be applied as a pre-specified fallback. Operationally, the full analysis set (FAS) will be used, defined as all randomized patients who have at least one valid post-randomization assessment of the primary outcome. The PPS, defined as patients who received protocolized EN for at least 48 h, will be used for sensitivity analyses.

Binary secondary outcomes (e.g., achievement of nutritional targets by day 7) will be analyzed analogously with GLMM (logit); continuous outcomes will use linear mixed models with a cluster random intercept and Kenward–Roger degrees-of-freedom adjustment. In addition, the EFI will be applied as a prespecified sensitivity analysis of the primary outcome to assess the robustness of the findings.

Continuous variables will be summarized as mean ± standard deviation if normally distributed, or as median and interquartile range (IQR, P25–P75) if not normally distributed. Between-group differences in continuous variables will be tested using the independent samples *t*-test for normally distributed data or the Mann–Whitney *U*-test for non-normally distributed data. Categorical variables will be compared using the Chi-square test.

Sensitivity analyses will include: (i) an unadjusted GLMM (treatment + random intercept only); (ii) a covariate-enriched GLMM that adds baseline mechanical ventilation and sepsis (yes/no; protocol-defined) on top of the pre-specified covariates (APACHE II score, AGI grade, primary diagnosis); and (iii) aFI. All covariates are pre-specified and retained regardless of statistical significance. We will report adjusted odds ratios (95% CIs) for binary endpoints (with marginal risk differences via standardization as supportive estimates). The primary endpoint will be tested at two-sided α = 0.05 without multiplicity adjustment. For prespecified clinical secondary endpoints, we will control the false discovery rate at *q* = 0.05 using the Benjamini–Hochberg procedure (two-sided *p*-values from the specified models). Process outcomes (fidelity, exposure, contamination) are exploratory and will be summarized descriptively with 95% CIs; no hypothesis testing or multiplicity adjustment will be applied to these measures. All analyses will be performed using SAS software version 9.4^®^ (SAS Institute Inc., Cary, NC, United States).

#### 2.7.2 Missing data and interpolation strategies

We will assume missing at random and use multivariate imputation by chained equations (MICE) with fully conditional specification in SAS 9.4, generating 50 imputed datasets and combining estimates via Rubin’s rules. The imputation model will include treatment arm and cluster identifier (ICU) to preserve clustering, plus pre-specified covariates.

#### 2.7.3 Confidentiality and data security

All data collected from participating centers will be stored in a secure web-based database. Except for the data administrator (the designated research lead at the coordinating center), no other individual will have the authority to modify, alter, or export patient data or information. Strict measures will be taken to ensure the confidentiality and security of all patient information. An independent Data and Safety Monitoring Board (DSMB)—separate from the sponsor, investigators, and coordinating center—will oversee participant safety and trial conduct under a written charter. An independent statistician will prepare unblinded safety summaries for the DSMB at prespecified intervals; the investigative team will remain blinded. The DSMB may issue recommendations (continue, modify, pause, or terminate) based on predefined safety considerations. Strict measures are in place to ensure confidentiality and data security throughout the study.

### 2.8 Adverse events

Adverse events are defined according to the CTCAE by the U.S. National Cancer Institute as any unfavorable medical occurrence in a patient receiving the study intervention, which does not necessarily have a causal relationship with the treatment.

During EN administration, adverse events may include FI, regurgitation, aspiration, or infection. Patients will be closely monitored for FI and laboratory abnormalities. Symptomatic treatment will be provided as needed, and measures such as head-of-bed elevation will be taken to reduce the risk of aspiration.

## 3 Discussion

Developing objective, guideline-based feeding protocols may improve outcomes in critically ill patients. Two of the largest cluster-randomized controlled trials to date have investigated this approach—one conducted by Doig et al. across 27 ICUs in Australia and New Zealand (3), and the other by Ke et al. across 97 ICUs in China (4). Unfortunately, neither feeding protocol resulted in improved clinical outcomes for critically ill patients. This raises critical questions: Is the impact of nutritional interventions inherently limited, or did the implemented protocols fail to provide real benefit to patients? The reasons behind these negative results remain unclear.

Upon reviewing the data from both trials, we observed that the intervention protocols did not effectively reduce the incidence of FI compared to standard care. The association between FI and poor clinical outcomes in critically ill patients has been well documented in multiple studies (15, 16). This led us to a bold hypothesis: feeding strategies may influence clinical outcomes by reducing FI, suggesting that FI could be a potential mediator between nutritional intervention and patient prognosis.

Unlike the two previous cluster-randomized controlled trials, our study did not adopt mortality as the primary endpoint. Nutritional interventions represent only one component of the overall management of critically ill patients, and directly reducing mortality through nutritional strategies alone appears challenging. The prior two trials did not demonstrate a mortality benefit, nor did they show a reduction in the incidence of FI. Therefore, in the present study we selected FI as the primary endpoint, as it is one of the most clinically relevant indicators of nutritional implementation and has been shown to be associated with patient outcomes (15). Although we do not think the mortality of the mNEED group will be lower, we expect it to at least reduce the occurrence of FI, which might also be beneficial.

A recently proposed concept further supports this hypothesis: in early nutrition, “less is more (17, 18).” Full-dose early nutrition may not benefit, and could even harm, critically ill patients by contributing to overfeeding (18). Therefore, a more promising approach may be to minimize FI while maintaining a gradual feeding progression.

Previously, we developed a predictive model for FI in critically ill patients based on variables such as primary diagnosis, APACHE II score, and AGI grade. This model demonstrated good predictive performance and was externally validated. Based on this, we integrated the tool into a revised feeding protocol to explore whether individualized nutrition strategies could improve outcomes. We adopted a cluster-randomized design, as applying two feeding protocols within the same ICU would be impractical and potentially unreliable.

The primary outcome is the incidence of FI within 7 days of ICU admission. We hypothesize that the mNEED protocol will reduce the incidence of FI. Whether it will also improve other clinical outcomes remains to be determined.

### 3.1 Strengths and limitations

This study has several notable strengths. First, the intervention strategy in the experimental group incorporated an FI prediction model, allowing for a relatively individualized feeding approach tailored to each patient’s predicted risk of FI. This individualized element is an important advancement compared with the two previous cluster-randomized controlled trials, which lacked such prediction-based strategies. Second, we selected FI as the primary outcome, hypothesizing a clear mechanistic pathway: nutritional interventions may influence mortality primarily through their effect on FI. Directly targeting mortality as the primary endpoint is challenging, as mortality is influenced by numerous non-nutritional factors and may be too distal an outcome to capture the effect of feeding strategies. Third, rigorous data quality control was implemented: each participating center appointed a data supervisor responsible for auditing bedside feeding records, verifying protocol adherence, and submitting all ultrasound images and feeding records for centralized review. This process substantially minimized bias and ensured fidelity of the intervention. Fourth, by engaging nearly 100 centers across China, this trial helped to standardize nutritional practice nationwide. Given that intensivists are not always fully familiar with every aspect of nutrition guidelines, the trial facilitated broader implementation and dissemination of guideline-based nutritional care.

This study also has important limitations. First, although FI was chosen as the primary outcome, its definition remains debated. Multiple diagnostic criteria exist; in this trial we adopted the widely accepted and relatively objective 2012 ESICM definition for FI, and applied the EFI definition as a prespecified sensitivity analysis. Despite this limitation, we consider FI the most suitable mediator between nutritional interventions and mortality. Second, center effects across nearly 100 ICUs are difficult to fully control. Although we stratified hospitals by level (secondary vs. tertiary) and performed randomization with a fixed seed, heterogeneity may still exist within the same level of hospitals—an inherent limitation of cluster-randomized designs, but also one that reflects the real-world variation in nutritional practice across China (19). Third, the feeding protocol in the control group was not entirely objective. Although based on the 2023 ESPEN guideline, interpretation of some aspects (e.g., initial feeding rate) remained subjective, which may have introduced variability in control group practices. Fourth, despite standardized ultrasound training delivered by CCUSG-certified instructors, operator-dependent variability could not be completely eliminated. Therefore, the change in muscle mass between day 1 and day 7 may be a more robust measure than the absolute value at day 7.

## 4 Ethics and dissemination

### 4.1 Research ethics approval and consent to participate

This study has been approved by the Ethics Committee of the First Hospital of Jilin University (original version: 24k004-001; revised version: 24k004-002). Ethical approval from each participating center will be required prior to patient enrollment. The study will be conducted in accordance with the principles of the Declaration of Helsinki (current version, Fortaleza, Brazil, 2013) and relevant regulations governing medical research involving human subjects.

The trial has been registered with the Chinese Clinical Trial Registry (ChiCTR2400081581): https://www.chictr.org.cn/showproj.html?proj=219477 and with ClinicalTrials.gov (NCT06827275): https://clinicaltrials.gov/study/NCT06827275.

### 4.2 Confidentiality issues

Processes and safeguards will be in place to protect all information stored in the web-based system. Data access will be secure and restricted to authorized users. Network protections will prevent unauthorized access or transfer of data.

Data security is ensured through multiple measures: servers are protected by firewalls, physical access is restricted, and the server is used exclusively for database management. Each participating site will only have access to data from its own patients, not those from other sites in the mNEED trial. All patient data will be de-identified to protect privacy, and no personal identifiers will appear in any publication. Confidentiality will be maintained in accordance with applicable regulations.

### 4.3 Potential risks and benefits

Based on previously published studies, the NOFI predictive tool has demonstrated good clinical utility and net benefit. By identifying patients at high risk of developing FI early, preemptive interventions can be implemented, which may reduce the incidence of FI during the acute phase and potentially improve clinical outcomes in critically ill patients.

### 4.4 Dissemination policy

The sponsor and principal investigators at all participating ICUs will have full access to the study data upon completion of the trial. Researchers wishing to conduct *post hoc* analyses must submit a formal written proposal to the writing and publication committee for approval.

## Footnotes

1
https://www.chictr.org.cn/showproj.html?proj=219477

2
https://clinicaltrials.gov/study/NCT06827275

3
https://space.bilibili.com/1909634094/favlist?fid=3540474994&ftype=create

4
https://www.youtube.com/playlist?list=PLtjoXcGeUkfy4BmDYvKvRkS97w_EidkN3
