# Supplementary material for: Modifying feeding protocols in critically ill patients based on a predictive model of feeding intolerance: protocol for a multicenter cluster randomized controlled trial (the mNEED study)
Source: Front Med (Lausanne). 2025 Nov 6;12:1649983. doi: 10.3389/fmed.2025.1649983 (PMC12630114; doi:10.3389/fmed.2025.1649983)
Supplement: Supplementary file 1 [file Table_1.docx]

**Supplementary materials**


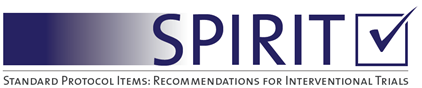


**Table S1: SPIRIT 2013 Checklist: Recommended items to address in a clinical trial protocol and related documents***

| Section/item | ItemNo | Description | Reported on page |  |
| --- | --- | --- | --- | --- |
| **Administrative information** | | |  |  |
| Title | 1 | Descriptive title identifying the study design, population, interventions, and, if applicable, trial acronym | 1 |  |
| Trial registration | 2a | Trial identifier and registry name. If not yet registered, name of intended registry | 2 |  |
|  | 2b | All items from the World Health Organization Trial Registration Data Set | 2 |  |
| Protocol version | 3 | Date and version identifier | 4 |  |
| Funding | 4 | Sources and types of financial, material, and other support | 23 |  |
| Roles and responsibilities | 5a | Names, affiliations, and roles of protocol contributors | 1 |  |
|  | 5b | Name and contact information for the trial sponsor | 1 |  |
|  | 5c | Role of study sponsor and funders, if any, in study design; collection, management, analysis, and interpretation of data; writing of the report; and the decision to submit the report for publication, including whether they will have ultimate authority over any of these activities | 23 |  |
|  | 5d | Composition, roles, and responsibilities of the coordinating centre, steering committee, endpoint adjudication committee, data management team, and other individuals or groups overseeing the trial, if applicable (see Item 21a for data monitoring committee) | 23 |  |
| Introduction |  |  |  |  |
| Background and rationale | 6a | Description of research question and justification for undertaking the trial, including summary of relevant studies (published and unpublished) examining benefits and harms for each intervention | 3-4 |  |
|  | 6b | Explanation for choice of comparators | 3-4 |  |
| Objectives | 7 | Specific objectives or hypotheses | 4 |  |
| Trial design | 8 | Description of trial design including type of trial (eg, parallel group, crossover, factorial, single group), allocation ratio, and framework (eg, superiority, equivalence, noninferiority, exploratory) | 4 |  |
| Methods: Participants, interventions, and outcomes | | |  |  |
| Study setting | 9 | Description of study settings (eg, community clinic, academic hospital) and list of countries where data will be collected. Reference to where list of study sites can be obtained | 4 |  |
| Eligibility criteria | 10 | Inclusion and exclusion criteria for participants. If applicable, eligibility criteria for study centres and individuals who will perform the interventions (eg, surgeons, psychotherapists) | 5 |  |
| Interventions | 11a | Interventions for each group with sufficient detail to allow replication, including how and when they will be administered | 7-8 |  |
|  | 11b | Criteria for discontinuing or modifying allocated interventions for a given trial participant (eg, drug dose change in response to harms, participant request, or improving/worsening disease) | Not applicable |  |
|  | 11c | Strategies to improve adherence to intervention protocols, and any procedures for monitoring adherence (eg, drug tablet return, laboratory tests) | 12 |  |
|  | 11d | Relevant concomitant care and interventions that are permitted or prohibited during the trial | 5 |  |
| Outcomes | 12 | Primary, secondary, and other outcomes, including the specific measurement variable (eg, systolic blood pressure), analysis metric (eg, change from baseline, final value, time to event), method of aggregation (eg, median, proportion), and time point for each outcome. Explanation of the clinical relevance of chosen efficacy and harm outcomes is strongly recommended | 13-15 |  |
| Participant timeline | 13 | Time schedule of enrolment, interventions (including any run-ins and washouts), assessments, and visits for participants. A schematic diagram is highly recommended (see Figure) | 8 |  |
| Sample size | 14 | Estimated number of participants needed to achieve study objectives and how it was determined, including clinical and statistical assumptions supporting any sample size calculations | 5-6 |  |
| Recruitment | 15 | Strategies for achieving adequate participant enrolment to reach target sample size | 5-6 |  |
| **Methods: Assignment of interventions (for controlled trials)** | | |  |  |
| Allocation: |  |  |  |  |
| Sequence generation | 16a | Method of generating the allocation sequence (eg, computer-generated random numbers), and list of any factors for stratification. To reduce predictability of a random sequence, details of any planned restriction (eg, blocking) should be provided in a separate document that is unavailable to those who enrol participants or assign interventions | 6 |  |
| Allocation concealment mechanism | 16b | Mechanism of implementing the allocation sequence (eg, central telephone; sequentially numbered, opaque, sealed envelopes), describing any steps to conceal the sequence until interventions are assigned | 6 |  |
| Implementation | 16c | Who will generate the allocation sequence, who will enrol participants, and who will assign participants to interventions | 6 |  |
| Blinding (masking) | 17a | Who will be blinded after assignment to interventions (eg, trial participants, care providers, outcome assessors, data analysts), and how | 6-7 |  |
|  | 17b | If blinded, circumstances under which unblinding is permissible, and procedure for revealing a participant’s allocated intervention during the trial | 6-7 |  |
| **Methods: Data collection, management, and analysis** | | |  |  |
| Data collection methods | 18a | Plans for assessment and collection of outcome, baseline, and other trial data, including any related processes to promote data quality (eg, duplicate measurements, training of assessors) and a description of study instruments (eg, questionnaires, laboratory tests) along with their reliability and validity, if known. Reference to where data collection forms can be found, if not in the protocol | 17-19 |  |
|  | 18b | Plans to promote participant retention and complete follow-up, including list of any outcome data to be collected for participants who discontinue or deviate from intervention protocols | 17-19 |  |
| Data management | 19 | Plans for data entry, coding, security, and storage, including any related processes to promote data quality (eg, double data entry; range checks for data values). Reference to where details of data management procedures can be found, if not in the protocol | 19 |  |
| Statistical methods | 20a | Statistical methods for analysing primary and secondary outcomes. Reference to where other details of the statistical analysis plan can be found, if not in the protocol | 17-19 |  |
|  | 20b | Methods for any additional analyses (eg, subgroup and adjusted analyses) | 17-19 |  |
|  | 20c | Definition of analysis population relating to protocol non-adherence (eg, as randomised analysis), and any statistical methods to handle missing data (eg, multiple imputation) | 17-19 |  |
| **Methods: Monitoring** | | |  |  |
| Data monitoring | 21a | Composition of data monitoring committee (DMC); summary of its role and reporting structure; statement of whether it is independent from the sponsor and competing interests; and reference to where further details about its charter can be found, if not in the protocol. Alternatively, an explanation of why a DMC is not needed | Not applicable |  |
|  | 21b | Description of any interim analyses and stopping guidelines, including who will have access to these interim results and make the final decision to terminate the trial | Not applicable |  |
| Harms | 22 | Plans for collecting, assessing, reporting, and managing solicited and spontaneously reported adverse events and other unintended effects of trial interventions or trial conduct | 19 |  |
| Auditing | 23 | Frequency and procedures for auditing trial conduct, if any, and whether the process will be independent from investigators and the sponsor | Not applicable |  |
| Ethics and dissemination | | |  |  |
| Research ethics approval | 24 | Plans for seeking research ethics committee/institutional review board (REC/IRB) approval | 22 |  |
| Protocol amendments | 25 | Plans for communicating important protocol modifications (eg, changes to eligibility criteria, outcomes, analyses) to relevant parties (eg, investigators, REC/IRBs, trial participants, trial registries, journals, regulators) | Not applicable |  |
| Consent or assent | 26a | Who will obtain informed consent or assent from potential trial participants or authorised surrogates, and how (see Item 32) | 4 |  |
|  | 26b | Additional consent provisions for collection and use of participant data and biological specimens in ancillary studies, if applicable | Not applicable |  |
| Confidentiality | 27 | How personal information about potential and enrolled participants will be collected, shared, and maintained in order to protect confidentiality before, during, and after the trial | 17 |  |
| Declaration of interests | 28 | Financial and other competing interests for principal investigators for the overall trial and each study site | 24 |  |
| Access to data | 29 | Statement of who will have access to the final trial dataset, and disclosure of contractual agreements that limit such access for investigators | 24 |  |
| Ancillary and post-trial care | 30 | Provisions, if any, for ancillary and post-trial care, and for compensation to those who suffer harm from trial participation | Not applicable |  |
| Dissemination policy | 31a | Plans for investigators and sponsor to communicate trial results to participants, healthcare professionals, the public, and other relevant groups (eg, via publication, reporting in results databases, or other data sharing arrangements), including any publication restrictions | Not applicable |  |
|  | 31b | Authorship eligibility guidelines and any intended use of professional writers | Not applicable |  |
|  | 31c | Plans, if any, for granting public access to the full protocol, participant-level dataset, and statistical code | Not applicable |  |
| Appendices |  |  |  |  |
| Informed consent materials | 32 | Model consent form and other related documentation given to participants and authorised surrogates | Not applicable |  |
| Biological specimens | 33 | Plans for collection, laboratory evaluation, and storage of biological specimens for genetic or molecular analysis in the current trial and for future use in ancillary studies, if applicable | Not applicable |  |

*It is strongly recommended that this checklist be read in conjunction with the SPIRIT 2013 Explanation & Elaboration for important clarification on the items. Amendments to the protocol should be tracked and dated. The SPIRIT checklist is copyrighted by the SPIRIT Group under the Creative Commons “[Attribution-NonCommercial-NoDerivs 3.0 Unported](http://www.creativecommons.org/licenses/by-nc-nd/3.0/)” license.

**Table S2. CONSORT 2025 checklist**

| Section/topic | No | CONSORT 2025 checklist item description | Reported on page no. |
| --- | --- | --- | --- |
| **Title and abstract** | | |  |
| Title and structured abstract | 1a | Identification as a randomised trial | 1 |
|  | 1b | Structured summary of the trial design, methods, results, and conclusions | 2 |
| **Open science** | | |  |
| Trial registration | 2 | Name of trial registry, identifying number (with URL) and date of registration | 2 |
| Protocol and statistical analysis plan | 3 | Where the trial protocol and statistical analysis plan can be accessed | Not applicable |
| Data sharing | 4 | Where and how the individual de-identified participant data (including data dictionary), statistical code and any other materials can be accessed | Not applicable |
| Funding and conflicts of interest | 5a | Sources of funding and other support (eg, supply of drugs), and role of funders in the design, conduct, analysis and reporting of the trial | 23 |
|  | 5b | Financial and other conflicts of interest of the manuscript authors | 23 |
| **Introduction** | | |  |
| Background and rationale | 6 | Scientific background and rationale | 3-4 |
| Objectives | 7 | Specific objectives related to benefits and harms | 3-4 |
| **Methods** | | |  |
| Patient and public involvement | 8 | Details of patient or public involvement in the design, conduct and reporting of the trial | 4 |
| Trial design | 9 | Description of trial design including type of trial (eg, parallel group, crossover), allocation ratio, and framework (eg, superiority, equivalence, non-inferiority, exploratory) | 1 |
| Changes to trial protocol | 10 | Important changes to the trial after it commenced including any outcomes or analyses that were not prespecified, with reason | Not applicable |
| Trial setting | 11 | Settings (eg, community, hospital) and locations (eg, countries, sites) where the trial was conducted | 24 |
| Eligibility criteria | 12a | Eligibility criteria for participants | 4-5 |
|  | 12b | If applicable, eligibility criteria for sites and for individuals delivering the interventions (eg, surgeons, physiotherapists) | Not applicable |
| Intervention and comparator | 13 | Intervention and comparator with sufficient details to allow replication. If relevant, where additional materials describing the intervention and comparator (eg, intervention manual) can be accessed | 7-12 |
| Outcomes | 14 | Prespecified primary and secondary outcomes, including the specific measurement variable (eg, systolic blood pressure), analysis metric (eg, change from baseline, final value, time to event), method of aggregation (eg, median, proportion), and time point for each outcome | 13-15 |
| Harms | 15 | How harms were defined and assessed (eg, systematically, non-systematically) | 19 |
| Sample size | 16a | How sample size was determined, including all assumptions supporting the sample size calculation | 5-6 |
|  | 16b | Explanation of any interim analyses and stopping guidelines | 5-6 |
| Randomisation: |  |  |  |
| Sequence generation | 17a | Who generated the random allocation sequence and the method used | 6 |
|  | 17b | Type of randomisation and details of any restriction (eg, stratification, blocking and block size) | 6 |
|  |  |  | **Reported on page no.** |
| Allocation concealment mechanism | 18 | Mechanism used to implement the random allocation sequence (eg, central computer/telephone; sequentially numbered, opaque, sealed containers), describing any steps to conceal the sequence until interventions were assigned | 6 |
| Implementation | 19 | Whether the personnel who enrolled and those who assigned participants to the interventions had access to the random allocation sequence | 6-7 |
| Blinding | 20a | Who was blinded after assignment to interventions (eg, participants, care providers, outcome assessors, data analysts) | 7 |
|  | 20b | If blinded, how blinding was achieved and description of the similarity of interventions | 7 |
| Statistical methods | 21a | Statistical methods used to compare groups for primary and secondary outcomes, including harms | 17-19 |
|  | 21b | Definition of who is included in each analysis (eg, all randomised participants), and in which group | 17-19 |
|  | 21c | How missing data were handled in the analysis | 19 |
|  | 21d | Methods for any additional analyses (eg, subgroup and sensitivity analyses), distinguishing prespecified from post hoc | 19 |
| **Results** | | |  |
| Participant flow, including flow diagram | 22a | For each group, the numbers of participants who were randomly assigned, received intended intervention, and were analysed for the primary outcome | Not applicable |
|  | 22b | For each group, losses and exclusions after randomisation, together with reasons | Not applicable |
| Recruitment | 23a | Dates defining the periods of recruitment and follow-up for outcomes of benefits and harms | Not applicable |
|  | 23b | If relevant, why the trial ended or was stopped | Not applicable |
| Intervention and comparator delivery | 24a | Intervention and comparator as they were actually administered (eg, where appropriate, who delivered the intervention/comparator, how participants adhered, whether they were delivered as intended (fidelity)) | Not applicable |
|  | 24b | Concomitant care received during the trial for each group | Not applicable |
| Baseline data | 25 | A table showing baseline demographic and clinical characteristics for each group | Not applicable |
| Numbers analysed,  outcomes and estimation | 26 | For each primary and secondary outcome, by group:  ● the number of participants included in the analysis  ● the number of participants with available data at the outcome time point  ● result for each group, and the estimated effect size and its precision (such as 95% confidence interval)  ● for binary outcomes, presentation of both absolute and relative effect size | Not applicable |
| Harms | 27 | All harms or unintended events in each group | Not applicable |
| Ancillary analyses | 28 | Any other analyses performed, including subgroup and sensitivity analyses, distinguishing pre-specified from post hoc | Not applicable |
| **Discussion** | | |  |
| Interpretation | 29 | Interpretation consistent with results, balancing benefits and harms, and considering other relevant evidence | Not applicable |
| Limitations | 30 | Trial limitations, addressing sources of potential bias, imprecision, generalisability, and, if relevant, multiplicity of analyses | Not applicable |

Citation: Hopewell S, Chan AW, Collins GS, Hróbjartsson A, Moher D, Schulz KF, et al. CONSORT 2025 Statement: updated guideline for reporting randomised trials. BMJ. 2025; 388:e081123. <https://dx.doi.org/10.1136/bmj-2024-081123>
© 2025 Hopewell et al. This is an Open Access article distributed under the terms of the Creative Commons Attribution License (<https://creativecommons.org/licenses/by/4.0/>), which permits unrestricted use, distribution, and reproduction in any medium, provided the original work is properly cited.

*We strongly recommend reading this statement in conjunction with the CONSORT 2025 Explanation and Elaboration and/or the CONSORT 2025 Expanded Checklist for important clarifications on all the items. We also recommend reading relevant CONSORT extensions. See [www.consort-spirit.org](http://www.consort-spirit.org).

We assessed the robustness of the sample size assumptions to variation in the intracluster correlation coefficient (ICC) and unequal cluster sizes. Specifically, we varied ICC = 0.01, 0.03, 0.05, 0.10, 0.15, 0.20 and the coefficient of variation (CV) of cluster size = 0.00, 0.30, 0.50. For each ICC×CV scenario, we recalculated the design effect using


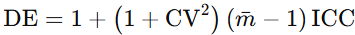


with planned average cluster size mˉ=25\bar m=25mˉ=25. Sample size targets were set at two-sided α=0.05\alpha=0.05α=0.05 and 90% power to detect an absolute reduction in FI from 0.50 (control) to 0.375 (intervention; 25% relative reduction). For every combination, we derived the required clusters per arm and additionally examined +10% cluster attrition. As a feasibility check, we also computed the achieved power when fixing 45 clusters per arm with mˉ=25\bar m=25mˉ=25 across the same grid of assumptions. Results are presented in Supplementary Table S1 (required clusters per arm) and Supplementary Table S2 (achieved power with 45 clusters/arm).

**Table S3. Required clusters per arm across ICC × CV scenarios (target power 90%).**

| ICC | CV of cluster size | Design effect (DE) | n per group if individual RCT | Inflated n per group (CRT) | Required clusters per arm | Clusters per arm with +10% cluster attrition |
| --- | --- | --- | --- | --- | --- | --- |
| 0.01 | 0.00 | 1.240 | 329 | 408 | 17 | 19 |
| 0.01 | 0.30 | 1.262 | 329 | 416 | 17 | 19 |
| 0.01 | 0.50 | 1.300 | 329 | 428 | 18 | 20 |
| 0.03 | 0.00 | 1.720 | 329 | 566 | 23 | 26 |
| 0.03 | 0.30 | 1.785 | 329 | 588 | 24 | 27 |
| 0.03 | 0.50 | 1.900 | 329 | 626 | 26 | 29 |
| 0.05 | 0.00 | 2.200 | 329 | 724 | 29 | 33 |
| 0.05 | 0.30 | 2.308 | 329 | 760 | 31 | 35 |
| 0.05 | 0.50 | 2.500 | 329 | 823 | 33 | 37 |
| 0.10 | 0.00 | 3.400 | 329 | 1119 | 45 | 50 |
| 0.10 | 0.30 | 3.616 | 329 | 1190 | 48 | 54 |
| 0.10 | 0.50 | 4.000 | 329 | 1316 | 53 | 59 |
| 0.15 | 0.00 | 4.600 | 329 | 1514 | 61 | 68 |
| 0.15 | 0.30 | 4.924 | 329 | 1620 | 65 | 73 |
| 0.15 | 0.50 | 5.500 | 329 | 1810 | 73 | 82 |
| 0.20 | 0.00 | 5.800 | 329 | 1909 | 77 | 86 |
| 0.20 | 0.30 | 6.232 | 329 | 2051 | 83 | 93 |
| 0.20 | 0.50 | 7.000 | 329 | 2303 | 93 | 104 |

**Table S4. Achieved power assuming 45 clusters per arm (m = 25) across ICC × CV scenarios.**

| ICC | CV of cluster size | Design effect (DE) | Effective n per group (with 45 clusters/arm) | Achieved power (two-sided α=0.05) |
| --- | --- | --- | --- | --- |
| 0.01 | 0.00 | 1.240 | 907.3 | 1.000 |
| 0.01 | 0.30 | 1.262 | 891.7 | 1.000 |
| 0.01 | 0.50 | 1.300 | 865.4 | 1.000 |
| 0.03 | 0.00 | 1.720 | 654.1 | 0.996 |
| 0.03 | 0.30 | 1.785 | 630.3 | 0.995 |
| 0.03 | 0.50 | 1.900 | 592.1 | 0.992 |
| 0.05 | 0.00 | 2.200 | 511.4 | 0.982 |
| 0.05 | 0.30 | 2.308 | 487.4 | 0.978 |
| 0.05 | 0.50 | 2.500 | 450.0 | 0.968 |
| 0.10 | 0.00 | 3.400 | 330.9 | 0.904 |
| 0.10 | 0.30 | 3.616 | 311.1 | 0.886 |
| 0.10 | 0.50 | 4.000 | 281.2 | 0.854 |
| 0.15 | 0.00 | 4.600 | 244.6 | 0.802 |
| 0.15 | 0.30 | 4.924 | 228.5 | 0.775 |
| 0.15 | 0.50 | 5.500 | 204.5 | 0.729 |
| 0.20 | 0.00 | 5.800 | 194.0 | 0.706 |
| 0.20 | 0.30 | 6.232 | 180.5 | 0.675 |
| 0.20 | 0.50 | 7.000 | 160.7 | 0.624 |

**Notes:** Assumptions: control FI risk p1 = 0.50, intervention FI risk p2 = 0.375, two-sided α = 0.05, target power = 0.90, average cluster size m = 25. Required clusters per arm were obtained by first computing the per-group sample size for an individually randomized trial (difference in proportions) and inflating by DE; the inflated per-group sample was then divided by m and rounded up. Achieved power with 45 clusters/arm was computed by converting to an effective per-group sample size n_eff = (45 × m)/DE and evaluating the approximate two-proportion test power under the alternative. CV denotes the coefficient of variation of cluster size; when CV = 0, clusters are equal-sized. All results are rounded to 3 decimal places where applicable.
